# Endothelin A receptor inhibition increases nitric oxide-dependent vasodilation independent of superoxide in non-Hispanic Black young adults

**DOI:** 10.1152/japplphysiol.00739.2022

**Published:** 2023-03-09

**Authors:** Casey G. Turner, Matthew J. Hayat, Caroline Grosch, Arshed A. Quyyumi, Jeffrey S. Otis, Brett J. Wong

**Affiliations:** ^1^Department of Kinesiology and Health, Georgia State University, Atlanta, Georgia, United States; ^2^Department of Population Health Sciences, School of Public Health, Georgia State University, Atlanta, Georgia, United States; ^3^Emory Clinical Cardiovascular Research Institute, Emory University School of Medicine, Atlanta, Georgia, United States

**Keywords:** endothelin-1, endothelium, human, microdialysis, nitric oxide

## Abstract

Young non-Hispanic Black adults have reduced microvascular endothelial function compared with non-Hispanic White counterparts, but the mechanisms are not fully elucidated. The purpose of this study was to investigate the effect of endothelin-1 A receptor (ET_A_R) and superoxide on cutaneous microvascular function in young non-Hispanic Black (*n* = 10) and White (*n* = 10) adults. Participants were instrumented with four intradermal microdialysis fibers: *1*) lactated Ringer’s (control), *2*) 500 nM BQ-123 (ET_A_R antagonist), *3*) 10 μM tempol (superoxide dismutase mimetic), and *4*) BQ-123 + tempol. Skin blood flow was assessed via laser-Doppler flowmetry (LDF), and each site underwent rapid local heating from 33°C to 39°C. At the plateau of local heating, 20 mM l-NAME [nitric oxide (NO) synthase inhibitor] was infused to quantify NO-dependent vasodilation. Data are means ± standard deviation. NO-dependent vasodilation was decreased in non-Hispanic Black compared with non-Hispanic White young adults (*P* < 0.01). NO-dependent vasodilation was increased at BQ-123 sites (73 ± 10% NO) and at BQ-123 + tempol sites (71 ± 10%NO) in non-Hispanic Black young adults compared with control (53 ± 13%NO, *P* = 0.01). Tempol alone had no effect on NO-dependent vasodilation in non-Hispanic Black young adults (63 ± 14%NO, *P* = 0.18). NO-dependent vasodilation at BQ-123 sites was not statistically different between non-Hispanic Black and White (80 ± 7%NO) young adults (*P* = 0.15). ET_A_R contributes to reduced NO-dependent vasodilation in non-Hispanic Black young adults independent of superoxide, suggesting a greater effect on NO synthesis rather than NO scavenging via superoxide.

**NEW & NOTEWORTHY** Endothelin-1 A receptors (ET_A_Rs) have been shown to reduce endothelial function independently and through increased production of superoxide. We show that independent ET_A_R inhibition increases microvascular endothelial function in non-Hispanic Black young adults. However, administration of a superoxide dismutase mimetic alone and in combination with ET_A_R inhibition had no effect on microvascular endothelial function suggesting that, in the cutaneous microvasculature, the negative effects of ET_A_R in non-Hispanic Black young adults are independent of superoxide production.

## Graphical abstract

**Figure:**
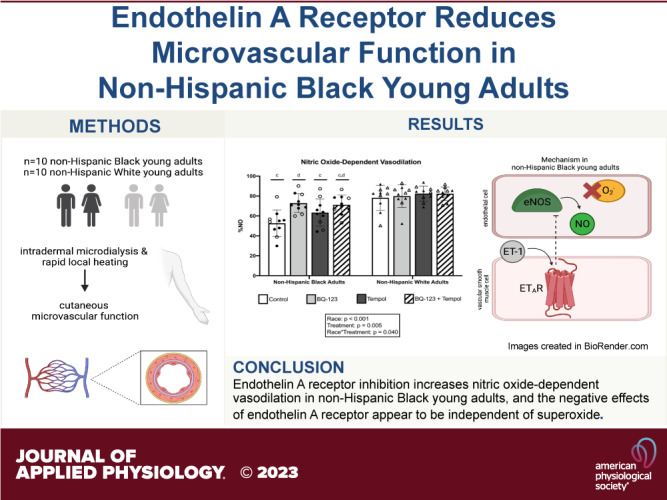

## INTRODUCTION

The balance between vasodilator and vasoconstrictor mechanisms is important for cardiovascular health (1). Across the lifespan, non-Hispanic Black adults often display an imbalance in these mechanisms, characterized by blunted vasodilator (2–14) and enhanced vasoconstrictor (15–20) responses, relative to non-Hispanic White adults (21). Likewise, the prevalence of cardiovascular disease (CVD) and several risk factors for CVD (i.e., hypertension, diabetes) is higher in non-Hispanic Black adults relative to non-Hispanic White adults (22). Understanding mechanisms underlying this disparity in vascular function is necessary to improve cardiovascular outcomes and clinical care in non-Hispanic Black adults across the lifespan.

There is a close interdependence between endothelial-derived nitric oxide (NO), a cardioprotective vasodilator (23), and endothelin-1 (ET-1), a powerful vasoconstrictor (24), often representing opposing mechanisms within the vasculature. However, ET-1 action depends on receptor subtype binding and location, where ET-1 binding to ET A (ET_A_R) or ET B (ET_B_R) receptor subtypes on vascular smooth muscle cells leads to vasoconstriction, but ET-1 binding to ET_B_R on endothelial cells yields vasodilation (25). Data from our laboratory and others have shown reduced microvascular endothelium- and NO-dependent vasodilation in healthy, non-Hispanic Black young adults relative to non-Hispanic White counterparts (2–6, 9). Increased ET-1 signaling is associated with reduced NO production and increased oxidative stress (26–30), in part through increased superoxide generation (24, 27). This phenotype is reflected in non-Hispanic Black adults across several ages and health statuses (2–6, 9, 10, 12, 31–34), and recent data (35) suggests inhibition of the ET_A_R can increase microvascular function in young, non-Hispanic Black and White women. However, it is currently unclear if ET_A_R inhibition influences microvascular function by affecting superoxide generation or if this mechanism is modified by self-identified racial background.

Microvascular endothelial function can be measured via local heating of the skin. Nonpainful, rapid heating of the skin elicits robust, biphasic, and reliable vasodilation (36–38). The initial vasodilation is a rapid, transient response mediated largely by sensory nerves and TRPV-1 channels with a modest contribution from NO (37, 39, 40). The second phase is a sustained plateau mediated largely (∼60%–80%) by endothelial NO-dependent mechanisms with contributions from other pathways, including endothelial-derived hyperpolarizing factors (EDHFs), adenosine receptors, and histamine receptors (37, 40–44). The ET_A_R subtype is functional in human skin and has been effectively inhibited during local heating (35, 45–49). Considering that cutaneous microvascular function can be used as a surrogate of systemic microvascular function (50), the present in vivo assessment of cutaneous microvascular vasodilation yields important context of the influence of ET-1 binding to the ET_A_R subtype and possible subsequent superoxide generation on vascular function in young, healthy non-Hispanic Black and non-Hispanic White adults.

The purpose of this study was to investigate the effect of ET_A_R antagonism, alone and in combination with a superoxide dismutase mimetic, on endothelium-dependent vasodilation and NO-dependent vasodilation in the cutaneous microvasculature of young non-Hispanic Black and White adults. We hypothesized that ET_A_R antagonism would increase endothelium-dependent vasodilation and NO-dependent vasodilation in non-Hispanic Black adults. We further hypothesized that combined inhibition of ET_A_R and superoxide would increase NO-dependent vasodilation to a greater extent than either treatment alone.

## METHODS

### Ethical Approval

This study was approved by Advarra Institutional Review Board (Columbia, MD; No. Pro00024265), the Georgia State University Institutional Review Board, and the United States Food and Drug Administration (IND 138231). All experimental procedures conformed with the Declaration of Helsinki. Each participant provided written and verbal informed consent before participating in any procedure.

### Participants

Participant characteristics are shown in Table 1. Participants who self-identified as either non-Hispanic Black (*n* = 10) or non-Hispanic White (*n* = 10) were recruited and tested. Women (*n* = 9 total) were tested during the menstrual phase of the natural menstrual cycle (*n* = 4) or during the placebo pill phase of oral contraceptives (OCP; *n* = 5). Menstrual cycle and oral contraceptive pill phase were determined by self-report and confirmed by cycle tracking via phone-based apps or presentation of pill pack. Women were required to submit a urine pregnancy test (McKesson hCG Combo Test Cassette, Consult Diagnostics; Richmond, VA) to confirm negative pregnancy status. Self-report health history and health habit information was obtained (Table 1). All participants were normotensive and were free of cardiovascular, pulmonary, and metabolic diseases and had no history of nerve pain/damage, cancer (or cancer treatment), or skin disorders (e.g., psoriasis). No participants used tobacco products, nicotine products, supplements, or medications (except for women using OCP). Further exclusion criteria included active COVID-19 infection, <1-mo post-COVID-19 infection via self-report date of positive test result, and long-lasting symptoms following known COVID-19 infection. Three participants reported a positive COVID-19 test (one non-Hispanic Black man, two non-Hispanic White women). All three indicated minor symptoms (cold-like symptoms, headache), and all three tested positive >10 mo before participation in this study. Recent data suggests there is little to no reduction in cutaneous NO-dependent vasodilation in young adults with mild-to-moderate COVID-19 within ∼1–8 mo after COVID-19 infection (51). Data from the participants who tested positive for COVID-19 were not the lowest in their respective cohort, suggesting mild COVID-19 did not affect their overall microvascular responses.

**Table 1.** Participant characteristics

	Non-Hispanic Black Young adults (*n* = 10)		Non-Hispanic White Young adults (*n* = 10)	
	Women (*n* = 4)	Men (*n* = 6)	Women (*n* = 5)	Men (*n* = 5)
Age, yr	22 ± 4	21 ± 2	21 ± 4	22 ± 3
Cycle information of women	2 NM		2 NM	
	2 OCP		3 OCP	
Height, m	1.64 ± 0.09	1.75 ± 0.05	1.61 ± 0.06	1.80 ± 0.05
Mass, kg	56.95 ± 10.86	71.30 ± 14.08	54.05 ± 5.43	81.88 ± 5.88
Body mass index, kg/m^2^	21.45 ± 4.58	23.28 ± 3.72	20.78 ± 2.07	25.42 ± 2.04
Systolic blood pressure, mmHg	112 ± 4	112 ± 7	112 ± 4	116 ± 4
Diastolic blood pressure, mmHg	69 ± 3	68 ± 3	69 ± 3	67 ± 6
Mean arterial pressure, mmHg	84 ± 3	83 ± 4	83 ± 3	83 ± 2
Heart rate, beats/min	67 ± 6	61 ± 5	64 ± 4	58 ± 9
Positive COVID-19 test (months before participating in the study)		1 (11 mo)	2 (10 and 11 mo)	
Physical activity, min/wk	133 ± 41	138 ± 25	130 ± 27	115 ± 49
Sleep, h/night	7.0 ± 1.4	7.2 ± 0.4	6.8 ± 0.5	7.3 ± 1.5
Alcohol, drinks/wk	0.5 ± 0.5	0.3 ± 0.5	1.2 ± 2.2	3.0 ± 2.6
Caffeine, drinks/wk	7 ± 8	2 ± 3	10 ± 8	13 ± 3

Data are means ± SD. NM, naturally menstruating; OCP, oral contraceptive pill.

### Instrumentation

Participants were asked to refrain from alcohol, vigorous exercise, and caffeine for at least 8 h before the experimental protocol. For the duration of the experiment, participants were seated in the semirecumbent position, and the experimental arm was positioned and secured at heart level to minimize the effect of hydrostatic pressure on perfusion pressure and, thus, blood flow. Participants were instrumented with four microdialysis fibers (CMA 31; Harvard Apparatus, Hollister, MA) on the dorsal forearm. The microdialysis fibers had a membrane 10 mm in length with a 55 kDa molecular weight cutoff. Microdialysis fibers were gas sterilized in ethylene oxide for 24-h using Georgia State University Core Facilities (Anprolene AN74 sterilizer and AN7916 gas kit; Andersen Sterilizers; Haw River, NC). An ice pack was used to numb the skin (52), and a 23-gauge needle was then placed into the dermal layer of the skin. The microdialysis fiber was threaded through the lumen of the needle, the microdialysis membrane was left in the dermal layer, and the needle was removed. Microdialysis sites were randomly assigned to receive *1*) lactated Ringer’s solution (Baxter Healthcare, Deerfield, IL) to serve as a control (53), *2*) 500 nM BQ-123 (54), an ET_A_R antagonist (AdipoGen Life Sciences, San Diego, CA), *3*) 10 μM tempol (5, 55–58), a superoxide dismutase mimetic (Sigma Aldrich, St. Louis, MO), or *4*) BQ-123 + tempol. All drugs were diluted in sterile lactated Ringer’s solution and drawn through sterile filter needles (BD Filter Needle; Becton Dickinson, Franklin Lakes, NJ) or sterile syringe filters (Acrodisc, 13 mm disk, 0.2 μm pore, hydrophilic PES membrane, USP Class VI; Pall Corporation, Port Washington, NY). Stock solutions of BQ-123 and tempol were prepared, separated, and stored in sterile vials at −20°C. Stock solutions were allowed to thaw to room temperature and protected from light before use. Stock solutions were kept for no longer than 30 days and each separate vial was only used once. Trauma from microdialysis fiber placement was allowed to resolve (∼45–60 min), and drugs were infused for at least 30 min before the experimental protocol at a rate of 2 μL/min (Beehive Controller and Baby Bee syringe pumps; Bioanalytical Systems, West Lafayette, IN).

To control local skin temperature, local heater units (VHP1 heater units and VMS-HEAT controller; Moor Instruments, Axminster, UK) were placed directly over each microdialysis membrane. Integrated laser-Doppler probes (VP7b probes and VMS-LDF2 monitor; Moor Instruments) were placed in the center of the local heating unit to obtain red blood cell flux, an index of skin blood flow, at each microdialysis site. Blood pressure was measured from the contralateral (right) arm using an automated brachial oscillometric device, and heart rate was derived from pulse detection (Welch Allyn Vital Signs Series 6000; Skaneatelles Falls, NY). Blood pressure and heart rate measurements were made every 10 min and mean arterial pressure (MAP) was calculated as one-third pulse pressure plus diastolic pressure.

### Experimental Protocol

Local heater units were first set at a thermoneutral temperature (33°C), and baseline skin blood flow was assessed for 10–15 min. Following baseline measurements, local heater temperature was increased from 33°C to 39°C at a rate of 0.1°C/s (59). No participants reported pain sensation during the local heating protocol. Once a plateau in skin blood flow was achieved (∼30–40 min into local heating), 20 mM *N*^ω^-nitro-l-arginine methyl ester (l-NAME; NO synthase inhibitor) was perfused through all microdialysis sites to quantify the percent contribution of NO to vasodilation (2, 4, 39, 60). When a plateau to l-NAME (i.e., post-l-NAME plateau) was achieved at all sites (∼30 min into l-NAME infusion), maximal vasodilation was induced by heating the skin from 39°C to 43°C (0.1°C/s) and infusing 54 mM sodium nitroprusside (SNP; NO donor and endothelium-independent vasodilator) (2–4, 60).

### Data Analysis

Skin blood flow data were continuously recorded at 40 Hz using commercially available hardware and software (PowerLab 16/35 data acquisition and Lab Chart 8 software; ADInstruments, Colorado Springs, CO). Cutaneous vascular conductance (CVC) was calculated as red blood cell flux divided by MAP and standardized to site-specific maximal vasodilation (%CVC_max_). Skin blood flow data used for analysis were averaged over a 3-min window as follows: *1*) baseline immediately preceding the onset of local heating, *2*) local heating plateau immediately preceding the infusion of l-NAME, *3*) post-l-NAME plateau immediately preceding initiation of maximal vasodilation, and *4*) maximum immediately preceding the end of the protocol. We also quantified NO-dependent vasodilation at all sites using the following equation: [(plateau magnitude – post-l-NAME plateau magnitude)/(plateau magnitude)] × 100 (39).

### Statistical Analysis

Sample size was determined with an a priori power analysis. Effect sizes were specified based on preliminary data from pilot studies completed in our laboratory. Assuming an α level of 0.05, 90% power, and mean %NO-dependent vasodilation of 52% (SD ± 12%) and 75% (SD ± 14%) for non-Hispanic Black and White young adults, respectively, the required sample size to detect this difference in means is 8 per group. Because the standard deviation (SD) from pilot data may not accurately reflect the SD from the study population, we increased our sample size by 25%. Thus, our final sample size was 10 participants per group. This sample size is similar to a recent study investigating the effect of BQ-123 on cutaneous NO-dependent vasodilation in non-Hispanic Black and White young women (35). Skin blood flow (%CVC_max_) and %NO-dependent vasodilation data were analyzed using a general linear model (i.e., two-way analysis of variance) with factors for racial identity (non-Hispanic Black and non-Hispanic White) and microdialysis treatment (control, BQ-123, tempol, and BQ-123 + tempol). Tukey’s post hoc test was used to estimate pairwise comparisons. All data were analyzed and graphed using commercially available software (SAS, Cary, NC and GraphPad Prism 8, San Diego, CA). The level of significance was set at *P* ≤ 0.05. All data are presented as means ± SD.

## RESULTS

Baseline, post-l-NAME plateau, and maximal CVC data are shown in Table 2. There was a main effect of race for maximal CVC (*P* = 0.01), but there were no other significant main or interaction effects (*P* > 0.2 for all).

**Table 2.** Baseline, post-l-NAME, and maximal skin blood flow data

	Non-Hispanic Black Young Adults (*n* = 10)	Non-Hispanic White Young Adults (*n* = 10)


Baseline, %CVC_max_		
Control	13 ± 5	13 ± 6
BQ-123	21 ± 9	11 ± 5
Tempol	18 ± 10	15 ± 8
BQ-123 + Tempol	22 ± 8	11 ± 5
Post-l-NAME, %CVC_max_		
Control	22 ± 9	16 ± 9
BQ-123	17 ± 8	17 ± 12
Tempol	19 ± 11	14 ± 5
BQ-123 + Tempol	18 ± 7	15 ± 5
Maximal, CVC*		
Control	2.20 ± 0.96	2.03 ± 0.70
BQ-123	2.32 ± 0.54	1.99 ± 0.64
Tempol	2.63 ± 0.47	1.94 ± 0.97
BQ-123 + Tempol	2.72 ± 0.67	2.05 ± 0.83

Data are means ± SD. Baseline and post-l-NAME values are %CVC_max_ and maximal values are CVC (flux/mmHg). *There was a main effect of race (*P* = 0.01) for maximal CVC such that maximal CVC was higher in non-Hispanic Black young adults. Data were analyzed using a two-way ANOVA with factors of self-identified racial identity and treatment site. BQ-123, endothelin A receptor antagonist; CVC, cutaneous vascular conductance; %CVC_max_, percent of site-specific maximal vasodilation; l-NAME, *N*^ω^-nitro-l-arginine methyl ester; Tempol, superoxide dismutase mimetic.

### Plateau Data

**Figure 1.**
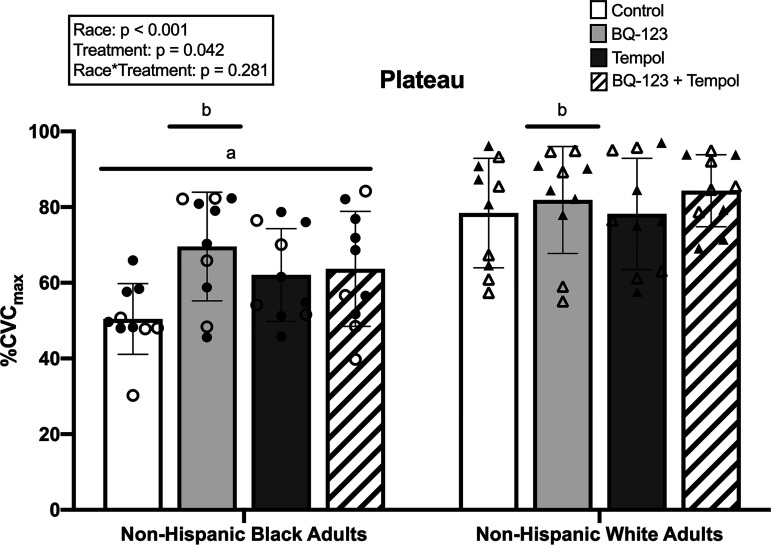
Endothelium-dependent vasodilation (i.e., plateau) between non-Hispanic Black (*n* = 10 participants) and non-Hispanic White (*n* = 10 participants) young adults. Data are presented as means ± SD. Circles represent non-Hispanic Black young adults. Triangles represent non-Hispanic White young adults. Open symbols, women. Closed symbols, men. Data were analyzed using a two-way ANOVA with factors of self-identified racial identity and treatment site. Symbols of significance represent main effects. ^a^*P* ≤ 0.05 compared with all sites in non-Hispanic White young adults. ^b^*P* ≤ 0.05 compared with control sites in the overall cohort. Exact *P* values are provided in the main text. BQ-123, endothelin A receptor antagonist; Tempol, superoxide dismutase mimetic.

Plateau data (i.e., endothelium-dependent vasodilation) is shown in Fig. 1. In non-Hispanic Black young adults, plateau was 50 ± 9%CVC_max_ at control sites, 70 ± 14%CVC_max_ at BQ-123 sites, 62 ± 12%CVC_max_ at tempol sites, and 64 ± 15%CVC_max_ at BQ-123 + tempol sites. In non-Hispanic White young adults, plateau was 78 ± 14%CVC_max_ at control sites, 82 ± 14%CVC_max_ at BQ-123 sites, 78 ± 15%CVC_max_ at tempol sites, and 84 ± 10%CVC_max_ at BQ-123 + tempol sites. There was not a significant interaction effect of race × treatment for plateau data (*P* = 0.28). However, there were significant main effects of self-identified race (*P* < 0.01) indicating the plateau was lower in non-Hispanic Black young adults relative to non-Hispanic White young adults (*P* < 0.01). There was also a significant main effect of treatment (*P* = 0.04) indicating ET_A_R inhibition with BQ-123 increased the plateau in the entire cohort compared with plateau at control sites in the entire cohort (*P* = 0.05).

### NO-Dependent Vasodilation

**Figure 2.**
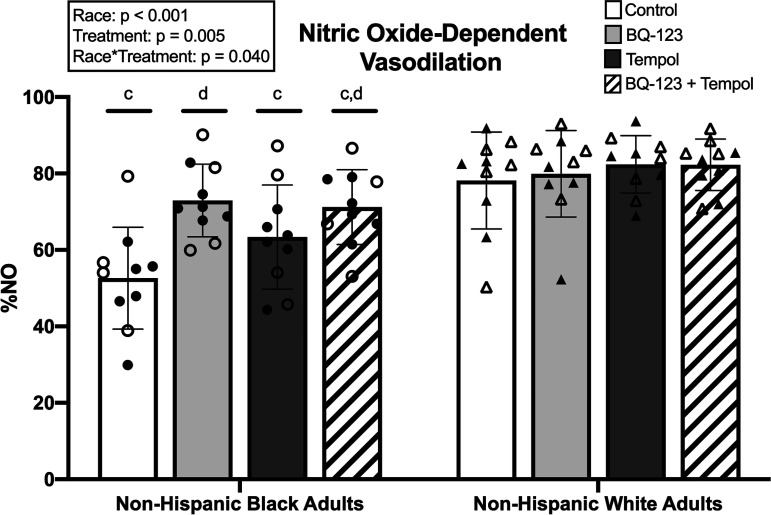
Nitric oxide (NO)-dependent vasodilation between non-Hispanic Black (*n* = 10 participants) and non-Hispanic White (*n* = 10 participants) young adults. Data are presented as means ± SD. Circles represent non-Hispanic Black young adults. Triangles represent non-Hispanic White young adults. Open symbols, women. Closed symbols, men. Data were analyzed using a two-way ANOVA with factors of self-identified racial identity and treatment site. Tukey’s post hoc test was used to assess pairwise comparisons. Symbols of significance represent post hoc analyses of the interaction effect. ^c^*P* ≤ 0.05 compared with respective treatment site in non-Hispanic White young adults. ^d^*P* ≤ 0.05 compared with control site within group. Exact *P* values are provided in the main text. BQ-123, endothelin A receptor antagonist; Tempol, superoxide dismutase mimetic.

NO-dependent vasodilation is shown in Fig. 2. In non-Hispanic Black young adults, NO-dependent vasodilation was 53 ± 13% NO at control sites, 73 ± 10% NO at BQ-123 sites, 63 ± 14% NO at tempol sites, and 71 ± 10% NO at BQ-123 + tempol sites. In non-Hispanic White young adults, NO-dependent vasodilation was 78 ± 13% NO at control sites, 80 ± 11% NO at BQ-123 sites, 82 ± 7% NO at tempol sites, and 82 ± 7% NO at BQ-123 + tempol sites. There was a main effect of racial identity (*P* < 0.01), where NO-dependent vasodilation overall was lower in non-Hispanic Black relative to non-Hispanic White young adults. There was also a main effect of treatment (*P* < 0.01), where BQ-123 perfusion, alone or in combination with tempol, increased NO-dependent vasodilation in the entire cohort compared with control sites. Furthermore, there was a significant interaction effect of race × treatment for NO-dependent vasodilation (*P* = 0.04). NO-dependent vasodilation was lower in non-Hispanic Black relative to non-Hispanic White young adults at control (*P* < 0.01), tempol (*P* < 0.01), and BQ-123 + tempol sites (*P* = 0.03), but not at BQ-123 sites alone (*P* = 0.15). In non-Hispanic Black young adults, BQ-123 (*P* < 0.01) and BQ-123 + tempol (*P* < 0.01) increased NO-dependent vasodilation compared with respective control site, whereas tempol alone did not have a significant effect (*P* = 0.18). In non-Hispanic White young adults, no treatment significantly affected NO-dependent vasodilation.

## DISCUSSION

The main findings of this study are as follows: *1*) ET_A_R antagonism increased endothelium-dependent vasodilation (plateau; Fig. 1) overall in this cohort of young, normotensive adults independent of self-identified racial group, *2*) ET_A_R inhibition increased the percent contribution of NO in non-Hispanic Black young adults to a level not different than that observed in non-Hispanic White young adults (Fig. 2), and *3*) ET-1 signaling through ET_A_R appears to affect endothelium-dependent vasodilation and NO-dependent vasodilation independent of superoxide in young, normotensive non-Hispanic Black adults.

### Endothelium-Dependent Vasodilation and NO-Dependent Vasodilation

Results from the present study are consistent with previous data showing decreased endothelium-dependent microvascular vasodilation in young, healthy non-Hispanic Black adults relative to non-Hispanic White counterparts (2–6, 9). Neither administration of an ET_A_R antagonist (BQ-123) nor a superoxide dismutase mimetic (tempol) influenced endothelium-dependent vasodilation uniquely in either group, but ET_A_R antagonism did increase endothelium-dependent vasodilation in the overall cohort (Fig. 1). Despite a ∼40% increase in the magnitude of endothelium-dependent vasodilation in non-Hispanic Black young adults with ET_A_R antagonism, there was also greater variability within the response than at control sites. However, there was a greater observed maximal CVC in non-Hispanic Black compared with non-Hispanic White young adults (Table 2), and this may suggest augmented vascular smooth muscle function in non-Hispanic Black young adults, perhaps to compensate for lower endothelial function. However, this response to nitroprusside may depend on the size of the blood vessel and/or the vascular bed (e.g., muscle vs. skin) as some data have shown a reduced forearm blood flow response to nitroprusside in non-Hispanic Black adults (10).

Findings from the present study are also consistent with previous data indicating decreased contribution of NO to endothelium-dependent microvascular vasodilation in young, healthy non-Hispanic Black relative to non-Hispanic White adults (2–6, 9) (Fig. 2). ET_A_R antagonism increased the percentage of NO-dependent vasodilation in non-Hispanic Black young adults to a level similar to that observed in non-Hispanic White young adults at control sites (Fig. 2). This could indicate that ET-1 signaling through ET_A_R does not uniquely impact the overall capacity of the endothelium to elicit dilation in young, normotensive non-Hispanic Black adults but instead regulates changes in the underlying mechanisms.

The present data also suggest that when using local heating of the skin to characterize endothelium-dependent vasodilator mechanisms and to interpret conclusions about NO-dependent vasodilation, it is important to inhibit NO synthase and directly quantify the contribution of NO to vasodilation, as a change (or lack thereof) in the plateau may not coincide with a change in NO-dependent vasodilation in magnitude or direction. Several pathways are known to contribute to the local heating plateau, and although NO is a major contributor, EDHFs have also been shown to make a significant contribution (∼15%–30%) to the plateau (59, 61). Previous work suggests EDHF mechanisms are preserved, but NO mechanisms are reduced, in healthy, middle-aged, Black adults compared with White counterparts (10). Therefore, it is possible EDHF mechanisms compensate for reductions in NO within healthy non-Hispanic Black young adults as well, and that this contribution from EDHF is amended when NO bioavailability increases during ET_A_R antagonism.

ET-1 signaling through the ET_A_R subtype is implicated in the pathogenesis of vascular dysfunction (62, 63), and Black adults have additional alterations in the endothelin system that may contribute to vascular dysfunction and disease. Hypertensive non-Hispanic Black adults exhibit greater ET_A_R subtype-dependent vasoconstriction compared with hypertensive non-Hispanic White counterparts (62). Black adults with diagnosed CVD show greater ET-converting enzyme protein levels than White counterparts (64). Furthermore, young non-Hispanic Black men have greater resting plasma ET-1 levels and greater ET-1 generation in response to acute psychological and physiological stressors compared with young non-Hispanic White men (65). As such, the present data support a mechanism for ET_A_R in blunted vasodilator responses in young, normotensive non-Hispanic Black adults. However, as this study was designed to antagonize the ET_A_R receptor, and not to decrease ET-1 itself, it is possible that ET_A_R antagonism allowed greater ET-1 signaling through endothelial ET_B_R, which mediates vasodilation and serves as an ET-1 clearance mechanism (45, 46, 49). In this subset of participants, ET_A_R appeared to have a greater effect on NO mechanisms. As NO is cardioprotective, these findings further suggest that ET-1 signaling through ET_A_R may be an optimal early intervention strategy to preserve, or restore, NO mechanisms in non-Hispanic Black young adults with early onset cardiovascular risk or disease.

From the present data, it is unclear how ET-1 receptor subtype expression may contribute to microvascular function in normotensive non-Hispanic Black young adults. ET-1 receptor subtype expression is altered in various conditions, such as hypertension (63, 64), coronary artery disease (66), and diabetes (67); however, changes in receptor expression appear to differ across these conditions. A greater vasoconstrictor phenotype appears to rely more on relative expression of ET_A_R to ET_B_R and cell type distribution than a generalized increase or decrease in ET_A_R expression alone (63, 64, 66, 67). Receptor subtype expression does appear to be independently related with ethnicity/identified race in disease states (64, 67), but this remains unknown in young, healthy individuals.

Previous data also suggest an influence of sex hormones on ET-1 receptor-mediated responses in women (45, 46, 48, 49, 54), and ET_A_R antagonism appears to improve cutaneous microvascular endothelium-dependent and NO-dependent vasodilation in both young Black and White women (35). Furthermore, there appear to be mechanistic differences in the regulation of vascular function between non-Hispanic Black men and women (9, 68). However, ET-1 receptor-mediated vascular responses have not been directly investigated for sex differences within the cutaneous microvasculature, and it is currently unclear how sex and racial identity may interact to affect these responses. Although there did not appear to be stark sex differences within our sample (symbols in Figs. 1 and 2 delineated to represent men and women), this study was not powered to analyze sex differences. Nevertheless, a fully powered study exploring sex differences in the effect of ET_A_R in microvascular function in non-Hispanic Black and non-Hispanic White young adults is warranted.

### The Role of Superoxide

Some of the detrimental properties of ET-1 have been attributed to its propensity to increase superoxide production by activating nicotinamide adenine dinucleotide phosphate (NADPH) oxidase (NOX) and/or inducing eNOS uncoupling (24, 27). In the cutaneous microvasculature, superoxide contributes to reductions in NO-dependent vasodilation in non-Hispanic Black young adults relative to non-Hispanic White young adults (5). The present study included administration of a superoxide dismutase mimetic (tempol), both alone and in combination with ET_A_R antagonism, to assess the related and independent effects of superoxide within this cohort. Tempol alone did not change either endothelium-dependent or NO-dependent vasodilation compared with respective control sites in either group (Figs. 1 and 2). Furthermore, tempol + BQ-123 did not differ from BQ-123 alone sites for either endothelium-dependent or NO-dependent vasodilation in either group (Figs. 1 and 2). It is unclear why tempol had no effect in the present study. Our data are in contrast with data previously reported by Hurr et al. (5), which showed an increase in local heating plateau and NO contribution [reported as the difference (Δ) between the local heating plateau and the post-l-NAME plateau] with tempol in Black compared with White young adults. Of note, the present study reports calculated %NO-dependent vasodilation (equation in methods). However, the difference between the local heating plateau and the post-l-NAME plateau (i.e., Δ) at tempol sites in non-Hispanic Black young adults in our study (43 ± 13%CVC_max_) is similar to that reported for Black young adults in the Hurr et al. study (48 ± 25%CVC_max_) (5). Furthermore, the magnitude of the local heating plateau in non-Hispanic Black young adults in the present study at control (50 ± 9%CVC_max_) and tempol (62 ± 12%CVC_max_) sites are also similar to those of Black young adults in the study from Hurr et al. (5) (control: 47 ± 15% CVC_max_, tempol: 63 ± 27% CVC_max_). Therefore, minor differences in observed values between these studies may account for differing statistical outcomes, but a similar mechanistic foundation is suggested by both studies.

Previous findings suggest NOX and xanthine oxidase (XO) as major sources of superoxide generation in young non-Hispanic Black adults, though there may be differences between young Black men and women (9). Patik et al. (9) reported increases in local heating plateau and NO contribution (Δ) to cutaneous microvascular vasodilation in Black young adults with inhibition of NOX and XO. Patik et al. (9) also found that young Black men responded to NOX and XO inhibition whereas young Black women did not. Although the present study was not powered to determine sex differences, both young Black women (plateau: 30% increase from control, %NO: 14% increase from control) and young Black men (plateau: 11% increase from control, %NO: 19% increase from control) appeared to respond positively to tempol administration. Furthermore, Akins et al. (35) recently found ET_A_R antagonism with BQ-123 to increase plateau and NO contribution (Δ) in both Black and White young women. These data collectively suggest ET_A_R and superoxide affect microvascular endothelial function and NO-dependent vasodilation independently in non-Hispanic Black young adults. However, it is unclear from the present study whether ET-1 signaling through ET_A_R affects NO bioavailability more greatly via changes in NO synthesis and/or eNOS expression, phosphorylation, or coupling, or through NO scavenging via reactive oxygen species in normotensive, non-Hispanic Black young adults. Regardless, trends within our data further supports the necessity of direct investigation of sex differences in the effect of ET_A_R in Black young adults.

### Limitations

There are a few limitations to this study that warrant consideration. First, social determinants of health are known to affect physiological mechanisms and responses and could contribute to the observations in the present study. Recent data from Wolf et al. (69) showed that social economic status (SES) was lower in a cohort of young African American participants relative to European American participants. However, there was no significant association between SES and cutaneous vasodilation in response to local heating (69). We did not directly assess SES or other social determinants of health but recognize that differences in these variables could account for the observed physiology. We did assess some health habits (Table 1), but these were self-report measures and were only assessed on the day of the study. Nevertheless, these self-report measures indicate groups were well-matched for these select self-report health habits. To fully assess a potential association between social determinants of health and microvascular responses would require a substantially larger participant cohort. Second, we recruited participants who self-identified their racial identity as either non-Hispanic Black or non-Hispanic White, as genotyping participants was beyond the scope of this study. We excluded participants who reported multiracial/ethnic and Hispanic backgrounds, but, because we relied on self-identification, it is possible there were participants of multiracial/ethnic or Hispanic lineage. Third, we did not perform blood analyses for fasting blood glucose or lipid profile. All participants were normotensive, and no participants reported having type 1 or 2 diabetes, hypercholesterolemia, or dyslipidemia. Furthermore, the magnitude of responses in both groups was similar to results of previous studies where glucose and lipids were analyzed (5, 9). Therefore, it is unlikely that our findings are the result of altered glucose or lipid status; however, we cannot objectively conclude that no participants had altered glucose or lipid status.

### Conclusions

This study presents evidence of a role for ET_A_R in reduced NO-dependent vasodilation in young, normotensive non-Hispanic Black adults compared with non-Hispanic White adults. Furthermore, this study suggests that ET-1 signaling through ET_A_R affects NO-dependent vasodilation independent of superoxide in this sample. Therefore, as NO is cardioprotective, upregulated ET-1 signaling through ET_A_R may be an early characteristic of cardiovascular risk in non-Hispanic Black young adults. Likewise, ET_A_R may be a possible target intervention for non-Hispanic Black young adults with early onset cardiovascular disease or vascular dysfunction.

## DATA AVAILABILITY

Data will be made available on reasonable request.

## GRANTS

This study was funded by NIH grant R01 HL141205 (to B.J.W.).

## DISCLOSURES

No conflicts of interest, financial or otherwise, are declared by the authors.

## AUTHOR CONTRIBUTIONS

C.G.T., A.A.Q., J.S.O., and B.J.W. conceived and designed research; C.G.T. performed experiments; C.G.T., M.J.H., C.G., and B.J.W. analyzed data; C.G.T., M.J.H., C.G., and B.J.W. interpreted results of experiments; C.G.T. and M.J.H. prepared figures; C.G.T. drafted manuscript; C.G.T., M.J.H., C.G., A.A.Q., J.S.O., and B.J.W. edited and revised manuscript; C.G.T., M.J.H., C.G., A.A.Q., J.S.O., and B.J.W. approved final version of manuscript.
